# Editorial: Omics technologies and bioinformatic tools in probiotic research

**DOI:** 10.3389/fmicb.2025.1577852

**Published:** 2025-03-04

**Authors:** Alex Galanis, Konstantinos Papadimitriou, Gerard M. Moloney

**Affiliations:** ^1^Department of Molecular Biology and Genetics, Democritus University of Thrace, Alexandroupolis, Greece; ^2^Department of Food Science and Human Nutrition, Agricultural University of Athens, Athens, Greece; ^3^Department of Anatomy and Neuroscience, University College Cork, Cork, Ireland

**Keywords:** probiotics, multi-omics, metagenomics, transcriptomics, databases, bioinformatic tools, whole genome sequence, CRISPR-Cas

Probiotics are defined as live microorganisms that can promote intestinal and extra-intestinal health benefits when consumed in sufficient quantities (Hill et al., 2014). Several bifidobacteria, *Lactobacilli*, and *Enterococci* have been classified as probiotics due to their safety profile and health-promoting properties. These microorganisms are commonly found in various habitats, such as dairy and non-dairy fermented products, the mammalian gastrointestinal microbiota and the environment. For a new strain to be classified as a probiotic, a number of criteria should be fulfilled: resistance to gastrointestinal transit, lack of virulence and transmissible antibiotic resistance genes, and health-promoting activities (e.g., antimicrobial, immunomodulatory, and antioxidant). Regulatory agencies have established conventional microbiological assays to assess these phenotypes (FAO/WHO, 2001). In addition, high-throughput multi-omics approaches are now being used to complement existing methodologies and provide deeper molecular and cellular insights into probiotic-host interactions (Kiousi et al., 2021).

In the era of (meta)genomics, the availability of whole genome sequences (WGS) of probiotic strains has increased exponentially. The integration of the genomic element in probiotic studies supports the prediction of the safety and functional profile of a new strain. In addition, WGS is the “gold standard” for the taxonomic classification of new isolates into species due to its higher discriminatory power. Indeed, the increased availability of WGS facilitated the reclassification of the diverse emended *Lactobacillus* spp. into 25 genera based on shared ecological and metabolic properties (Zheng et al., 2020). Currently, EFSA requires WGS of microorganisms to be used in the food chain to monitor genes of concern (e.g., virulence factors, antibiotic resistance genes) (EFSA, 2024). In this context, genomic analyses supplemented with *in vitro* assays were performed by Wei et al. to evaluate the safety and functional traits of *Limosilactobacillus reuteri* A51, a strain previously isolated from yak yogurt. The strain was found to encode genes related to stress response, survival and attachment in the gastrointestinal tract, along with biosynthetic clusters for antimicrobial compounds and exopolysaccharides. The strain also exhibited increased tolerance to simulated gastrointestinal conditions, as well as antioxidant and antimicrobial activity. Wang et al. performed a similar analysis for *Lactiplantibacillus plantarum* HOM2217, a strain isolated from human milk, and examined its potential as an alternative treatment for obesity. The strain was shown to have cholesterol-removing activity *in vitro*. In addition, it could regulate lipid metabolism and inflammation, therefore contributing to the prevention of obesity in rats fed a high-fat diet (HFD). Similarly, *Enterococcus rotai* CMTB-CA6, a strain isolated from the medicinal plant *Cantella asiatica* by Kim et al., limited the growth of skin pathogens, induced commensal growth and increased dermal fibroblast viability *in vitro*. No transferable antimicrobial resistance genes or virulence factors were identified in the WGS of the strain, suggesting its safe use in skin care products.

In addition to probiotics derived from aerobic or microaerophilic environments, strictly anaerobic commensal microorganisms, most notably *Feacalibacterium prausnitzii* and *Akkermansia muciniphila* have been used as next-generation probiotics (O'Toole et al., 2017). These microorganisms are adapted to grow in the GI tract of the host and thus present an evolutionary advantage in the niche. Indeed, Vergalito et al. showed that *A. muciniphila* ATCC BAA-835 exhibited increased viability and higher adhesion capacity under simulated GI tract conditions compared to *Lacticaseibacillus rhamnosus* GG, one of the most well-studied probiotic strains. Genes for mucus-degrading enzymes and two mucus-binding proteins involved in the adhesion capacity of *A. muciniphila* were also detected in the strain genome.

Comparative genomic analysis has supported the identification of conserved antiphage responses in the bacterial genome. One of these adaptive systems is clustered regularly interspaced short palindromic repeats (CRISPR)—and CRISPR-associated (Cas) loci, which encode for a primitive acquired immunity system that patrols the cytoplasm and degrades phage-derived genomic sequences (Makarova et al., 2020). In the context of the food industry, phages can reduce cell growth and cause cell lysis, thereby significantly affecting the fermentation capacity of microbial strains, and the texture and aroma of the end product (Ranveer et al., 2024). In lactobacilli CRISPR-Cas arrays are distributed in a strain-specific manner. Indeed, Rostampour et al. found that Cas proteins are present in 22% of the available genomes of *Lp. plantarum* strains, with subtype II-A being the most common among the strains, followed by type I-E. Further analysis showed that subtype II-A could play a more active role in the defense capacity of the strains due to its larger repeat-spacer arrays. Of note, these spacers also appear to confer resistance to plasmid-mediated gene transfer, therefore presenting broader activity. It should be noted that CRISPR-Cas engineering is a leading method for the efficient manipulation of genomic sequences. Therefore, understanding the prevalence of these systems in lactobacilli can support the expansion of the lactobacilli bioengineering toolkit (Parvin and Sadras, 2024). Furthermore, comparative genomics can be utilized for the identification of conserved genes involved in antibiotic resistance. Aborode et al. applied this method to identify antibiotic resistance genes in *Escherichia coli* genomes. The structure of the gene products was predicted *in silico* and docking experiments were performed to evaluate the ability of phytochemicals to be effective inhibitors. Utilizing this approach, the authors confirmed that hesperidin, a flavanone glycoside derived from citrus fruit, exhibited favorable pharmacokinetics, good stability and plausible binding positions against resistant *E. coli* strains carrying the *MacB, gidB*, and *katG* genes.

In conclusion, this Research Topic provides valuable knowledge and information on the mechanisms of action and health-promoting activities of novel potential probiotic strains isolated from different sources, such as dairy products and environmental samples. It is clear that the field of probiotics is moving toward mechanistic studies to establish causative relationships between probiotic administration and host health. In this context, new databases, search engines, and bioinformatics pipelines have been developed to systematically collect and present the bulk of the data presented in these studies, to support their meaningful interpretation and visualization, and to facilitate comparative analysis and the selection of the most promising strains for further research. For example, Probio-ichnos is a manually curated, literature-derived database that collects and presents data for microorganisms with *in vitro* probiotic properties (Tsifintaris et al., 2024), MASI catalogs the interactions between microbiota and probiotics with active substances (Zeng et al., 2021), and ODRAP explores prebiotic activity (Guseva et al., 2020). Moreover, the machine learning platform iProbiotics supports the rapid identification of probiotic properties from WGS data (Sun et al., 2022), whereas ProbioMinServer is an integrated platform for evaluating the safety and functionality of potential probiotic strains (Liu et al., 2023). These databases, platforms, bioinformatics tools, and methodologies presented in this Research Topic can be used in combination to provide a holistic view of the interplay between probiotics, food and intestinal microbiota, leading to tailor-made, strain-, host-, and disease-specific probiotic supplements.
